# Identifying More Epidemic Clones during a Hospital Outbreak of Multidrug-Resistant *Acinetobacter baumannii*

**DOI:** 10.1371/journal.pone.0045758

**Published:** 2012-09-27

**Authors:** Matthieu Domenech de Cellès, Jérôme Salomon, Anne Marinier, Christine Lawrence, Jean-Louis Gaillard, Jean-Louis Herrmann, Didier Guillemot

**Affiliations:** 1 Unité de Pharmacoépidémiologie et Maladies Infectieuses, Institut Pasteur, Paris, France; 2 U657, Institut national de la santé et de la recherche médicale, Paris, France; 3 Paris 6: Univ. Pierre et Marie Curie, Cellule Pasteur UPMC, Paris, France; 4 Laboratoire Modélisation et Surveillance des Risques de Sécurité Sanitaire, Conservatoire National des Arts et Métiers, Paris, France; 5 Laboratoire de Microbiologie, Hôpital Raymond-Poincaré, Assistance Publique–Hôpitaux de Paris, Garches, France; 6 EA 3647, Université de Versailles–Saint-Quentin-en-Yvelines, Versailles, France; 7 EA 4499, Université de Versailles–Saint-Quentin-en-Yvelines, Versailles, France; 8 Unité Fonctionnelle de Santé Publique, Hôpital Raymond-Poincaré, Assistance Publique–Hôpitaux de Paris, Garches, France; Monash University, Australia

## Abstract

Infections caused by multidrug-resistant bacteria are a major concern in hospitals. Current infection-control practices legitimately focus on hygiene and appropriate use of antibiotics. However, little is known about the intrinsic abilities of some bacterial strains to cause outbreaks. They can be measured at a population level by the pathogen’s transmission rate, i.e. the rate at which the pathogen is transmitted from colonized hosts to susceptible hosts, or its reproduction number, counting the number of secondary cases per infected/colonized host. We collected data covering a 20-month surveillance period for carriage of multidrug-resistant *Acinetobacter baumannii* (MDRAB) in a surgery ward. All isolates were subjected to molecular fingerprinting, and a cluster analysis of profiles was performed to identify clonal groups. We then applied stochastic transmission models to infer transmission rates of MDRAB and each MDRAB clone. Molecular fingerprinting indicated that 3 clonal complexes spread in the ward. A first model, not accounting for different clones, quantified the level of in-ward cross-transmission, with an estimated transmission rate of 0.03/day (95% credible interval [0.012–0.049]) and a single-admission reproduction number of 0.61 [0.30–1.02]. The second model, accounting for different clones, suggested an enhanced transmissibility of clone 3 (transmission rate 0.047/day [0.018–0.091], with a single-admission reproduction number of 0.81 [0.30–1.56]). Clones 1 and 2 had comparable transmission rates (respectively, 0.016 [0.001–0.045], 0.014 [0.001–0.045]). The method used is broadly applicable to other nosocomial pathogens, as long as surveillance data and genotyping information are available. Building on these results, more epidemic clones could be identified, and could lead to follow-up studies dissecting the functional basis for variation in transmissibility of MDRAB lineages.

## Introduction

Infections caused by multidrug-resistant bacteria represent a major public health challenge. Results of a recent study revealed that more than half of emerging infectious diseases involved bacteria, the majority of which had evolved towards drug-resistance [1]. Within hospitals, misuse of antibiotics and failure to comply with strict hygiene measures have contributed to the spread of resistant bacterial strains. As a consequence, many nosocomial pathogens, e.g. methicillin-resistant *Staphylococcus aureus* (MRSA), vancomycin-resistant *Enterococcus*, extended-spectrum–beta-lactamase producing Enterobacteriaceae, have acquired multiple mechanisms to escape the action of antimicrobials, resulting in high rates of resistance within hospital settings [2]. This situation has raised concerns over possible therapeutic failures and the perspective of a post-antibiotic era [3]. Therefore, methods are required to prevent and control the in-hospital spread of multiresistant bacteria.

Rigorous hygiene and correct antibiotics use are the cornerstones of infection control within hospital settings. Indeed, numerous studies demonstrated the importance of compliance with barrier precautions and environmental measures to stop transmission chains between patients and healthcare workers [4], [5], [6] and reasonable antibiotics use to prevent the emergence and spread of resistant strains [7], [8]. However, little is known about the intrinsic abilities of some bacterial strains to persist within hospitals. At a population level, they could impact bacterial strains’ capacities to transmit from host to host, measured by the transmission rate, that is the rate at which the pathogen is transmitted from colonized hosts to susceptible hosts, and the reproduction number, counting the number of secondary cases per colonized/infected host [9], [10].

Recent molecular biology advances enable bacterial strains identification at the molecular level. These techniques, based on amplified fragment-length polymorphism and DNA sequencing of a part or the total bacterial genome, have opened a wide field of research [11]. Yet, it remains unclear how to use this genetic information for epidemiological purposes or public health decision-making, and especially for infection control strategies in hospitals [12].

Herein, we describe a model-based approach to investigate transmissibility differences between bacterial strains of the same genetic lineage, i.e. clones. We applied this method to data collected in a surgery ward during a 20-month surveillance period for carriage of multiresistant *Acinetobacter baumannii* (MDRAB), a Gram-negative bacterium responsible for numerous outbreaks worldwide and now a serious threat within hospitals [13], [14].

## Methods

### Ethics Statement

This study used observational data collected as part of systematic routine surveillance procedures in a university hospital ward, with an endemic level of multidrug-resistant bacteria. This surveillance protocol follows the official recommendations of the French Ministry of Health and the French Society for Hygiene (http://sante.gouv.fr/les-infections-nosocomiales-recommandations-aux-etablissements-de-soins.html) and was approved by the Nosocomial Infections Fighting Committee. All the patients receive general information about the hospital infection control strategy at admission. No more information than those collected by routine procedures was used; in particular, no additional individual data, biological collection or sample was required. Therefore, an ethics committee approval and patients’ informed consent were not required for this study.

### Dataset

Data were collected in the orthopedic septic surgery and bedsore surgery ward (treating mainly spinal cord injured patients) of the Raymond-Poincaré University Hospital (Garches, France), from 1 January 2008 to 31 August 2009. During the surveillance period, all admissions and discharges were recorded in a database. Ward policy was to swab (rectal swab, plus bedsores swabs, if present) every patient within 48 hours following admission and then weekly for MDRAB surveillance. Multiresistance was defined as resistance to at least 3 classes of antibiotics. The initial 25-bed capacity (15 rooms, 10 2-bed rooms and 5 single-bed rooms) was then reduced to 15 in November 2008 (15 single-bed rooms), as part of preventive control measures to the outbreak.

### Molecular Typing of Isolates

Fifty-six MDRAB clinical isolates were cultured overnight at 37°C on tryptic soy-agar plates, and colonies were suspended in DNA lysis buffer for genomic DNA extraction, as described by the manufacturer (Qiamp DNA Mini Kit, Qiagen®, Courtaboeuf, France). Extracted DNA was stored at –20°C until use. Rep-PCR amplification was performed using the commercialized *Acinetobacter* Diversilab kit (BioMérieux, Marcy l’Etoile, France) as recommended, and a DNA Engine® thermocycler (BioRad, Marnes-La-Coquette, France). Amplified products were then separated using a 12 kb DNA chip, with migration performed as recommended (Experion, BioRad). Electrophoresis patterns were analyzed using BioNumerics software (version 6.5; Applied Maths, Sint Martens Laten, Belgium) using the following tolerance settings: 1% optimization, and 1% position tolerance. Patterns were clustered using Dice’s coefficients and the unweighted-pair–group method using average linkages to rep-PCR types. The effect of grouping similar rep-PCR patterns together into a smaller number of rep-PCR types was examined using 80% relatedness cutoffs [15], [16].

### Transmission Metrics

To estimate the transmission capacities of MDRAB and each MDRAB clone, we formulated mathematical population-based transmission models, described below. For all models, two quantities were used to estimate transmissibility. First, the transmission rate, measuring the rate at which a pathogen is transmitted from colonized hosts to susceptible hosts in the ward. Second, the single-admission reproduction number, defined as the average number of secondary cases caused by a single ward admission of the primary case when other patients are susceptible. Although this parameter is related to the basic reproduction number R_0_, it has a distinct interpretation, counting only secondary cases arising during a single ward admission when R_0_ includes repeated admissions, therefore R_A_< R_0_ [17].

### Nonclonal Transmission Model

The first model was derived from the model proposed in [18], to evaluate the MDRAB cross-transmission level in the ward. Patients within the ward are in 2 mutually exclusive states: susceptible or MDRAB-colonized. Patients enter the ward in one of these states and exit via discharge. Susceptible patients are at risk of acquiring MDRAB from other colonized patients, at a rate depending on both the frequency of carriage in the ward (colonization pressure) and the transmission capacity of MDRAB. Transmission of MDRAB between patients is supposed to occur through contact with healthcare workers. Although environment can act as a secondary source of transmission, we did not include a possible environmental reservoir in the model (we defer the justification of this point to the discussion). A stochastic model was used to capture chance effects due to the small population of patients in the ward (Text S1).

**Table 1:** Epidemiological data observed during the study period.

Data	Value
Mean [range] daily number of patients	15 [8]–[23]
Study-period duration (days)	609
Total admissions, *n*	440
Identified colonized patients, *n*	56
Mean [range] monthly MDRAB prevalence (/100patient-days)	15 [0–56]
Discharge rate/day	
Susceptible patients	0.042
MDRAB-colonized patients	0.048
Clone 1-MDRAB–colonized	0.035
Clone 2-MDRAB–colonized	0.055
Clone 3-MDRAB–colonized	0.058

### Clonal Transmission Model

We extended the first model to integrate the data provided by molecular typing of isolates. For each clone, a 3-state stochastic transmission model was built, with patients partitioned into 1 of the following states: not MDRAB colonized, colonized with this clone, or colonized with a strain not belonging to this clone. Again, a stochastic model was used (Text S1).

**Figure 1:**
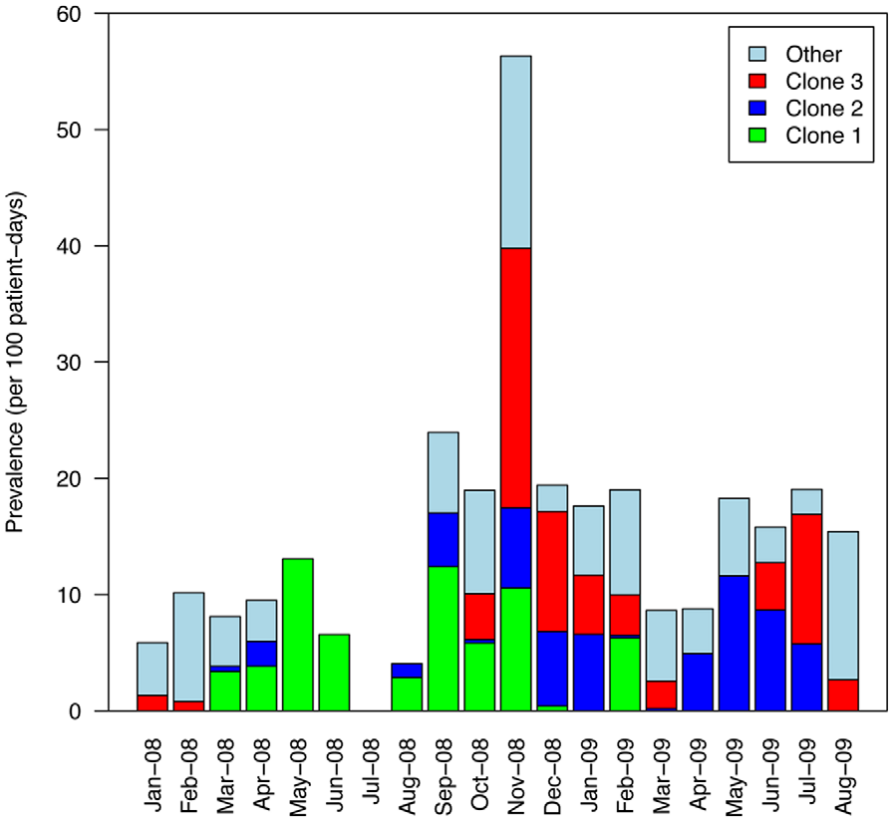
Monthly prevalence of MDRAB. Stacked bars represent the prevalence of patients colonized with clone 1 (green bar), clone 2 (blue bar), clone 3 (red bar) or other strains (light blue bar).

### Parameter Values

For both models, discharge rates were computed from surveillance data, using observed lengths of patients’ stays. For susceptible patients, the discharge rate was computed as 1/*los*, where *los* is the mean length of stay of susceptible patients; for patients colonized with MDRAB or a specific MDRAB clone, the discharge rate was taken as the reciprocal of the mean time from first identification to discharge. Because all patients were sampled within 48 hours following admission, their colonization status at admission was uncertain. Therefore, we estimated the proportion of carriers at admission. Both transmission metrics, namely the transmission rate and the single-admission reproduction number, were also estimated from the data. Specifically, the value of the single-admission reproduction number was computed as the transmission rate/discharge rate ratio, for MDRAB and each MDRAB clone.

**Table 2:** Parameter estimates (Mean [95% credible interval]) for MDRAB and each MDRAB clone.

Species/Clone	Importation probability	Transmission rate(per day)	Single-admission Reproduction number
MDRAB (total)	0.083 [0.032–0.149]	0.030 [0.012–0.049]	0.61 [0.26–1.02]
Clone 1 MDRAB	0.023 [0.008–0.048]	0.016 [0.001–0.045]	0.44 [0.03–1.30]
Clone 2 MDRAB	0.028 [0.012–0.052]	0.014 [0.001–0.045]	0.25 [0.02–0.81]
Clone 3 MDRAB	0.028 [0.012–0.054]	0.047 [0.018–0.091]	0.81 [0.30–1.56]

The single-admission reproduction number is defined as the average number of secondary cases caused by a single ward admission of the primary case when other patients are susceptible.

### Estimation Procedure

If exact colonization dates were known for all patients, maximization of the complete likelihood would provide direct estimates of the unknown parameters. However, because patients were only swabbed weekly, those dates were known only up to a censoring interval of 1 week. Therefore, a data-augmentation method is required to tackle this uncertainty. We implemented a Markov Chain Monte Carlo algorithm involving 2 steps: updating of parameters and data augmentation [19]. Details of the algorithm can be found in Text S1. All estimated parameters were assigned a diffuse Exp(0.001) prior; convergence of the chains was assessed using single-chain convergence tests of the R package coda [20]. All analyses were performed using R 2.12, free open-source statistical software [21].

### Model Checking

To assess model fit, we used the fitted transmission model to generate 1,000 simulated epidemic curves for carriage of MDRAB or each MDRAB clone, conditioned on patient colonization statuses on their first day and observed lengths of stay. Fitting was done in 2 steps. First, the epidemic curve was initialized with patients known to be colonized on their first day of stay. Second, the transmission model was applied to each remaining patient for each day of the study period. Finally, mean values and 95% credible intervals for the number of patients colonized with MDRAB or each MDRAB clone were computed and compared to the observed epidemic curves.

## Results

The dataset covered a 20-month surveillance period in a surgery ward, during which 350 patients had 440 admissions. Fifty-six patients had at least 1 positive MDRAB swab, and had 69 episodes of carriage. Other relevant epidemiological parameters and their values are listed in Table 1. Fig. 1 shows the monthly MDRAB prevalence during the study period.

**Figure 2:**
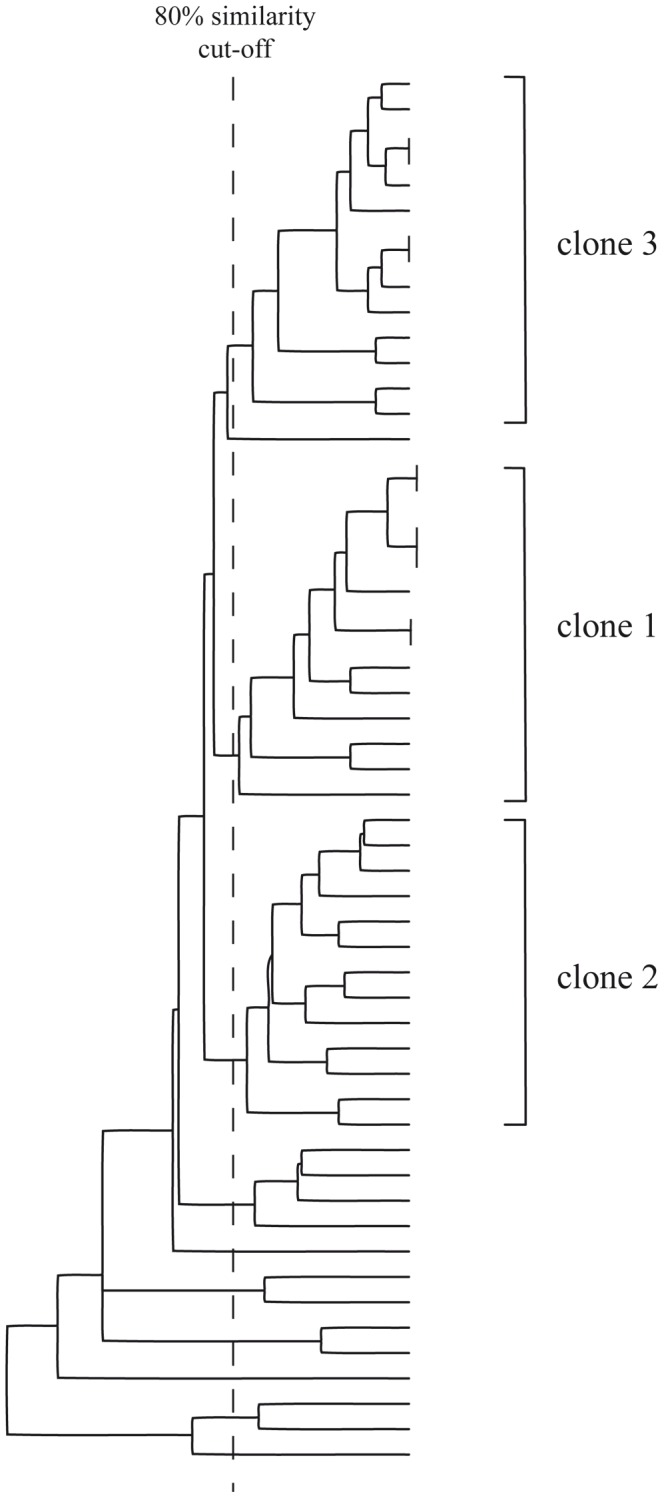
Phylogenetic tree of MDRAB isolates. Bionumerics® 6.5 was used to cluster rep-PCR profiles. An 80% similarity threshold was applied to form clusters.

From January 2008 to August 2008, MDRAB prevalence was low (mean: 7 per 100 patient-days, range (0–13)). From August 2008 until December 2008, the ward experienced a major MDRAB outbreak (mean prevalence: 30 per 100 patient-days, range (0.19–0.56)). Afterwards, MDRAB remained endemic in the ward, at a higher prevalence than before the outbreak (mean: 15 per 100 patient-days, range (0.09–0.19)).

From the nonclonal transmission model, the in-ward MDRAB transmission rate per day was estimated at 0.030 (95% credible interval [0.012–0.049]), corresponding to a single admission-reproduction number of 0.61 [0.26–1.02]. The proportion of carriers at admission was estimated to be 0.08 [0.03–0.15] (Table 2).

**Figure 3:**
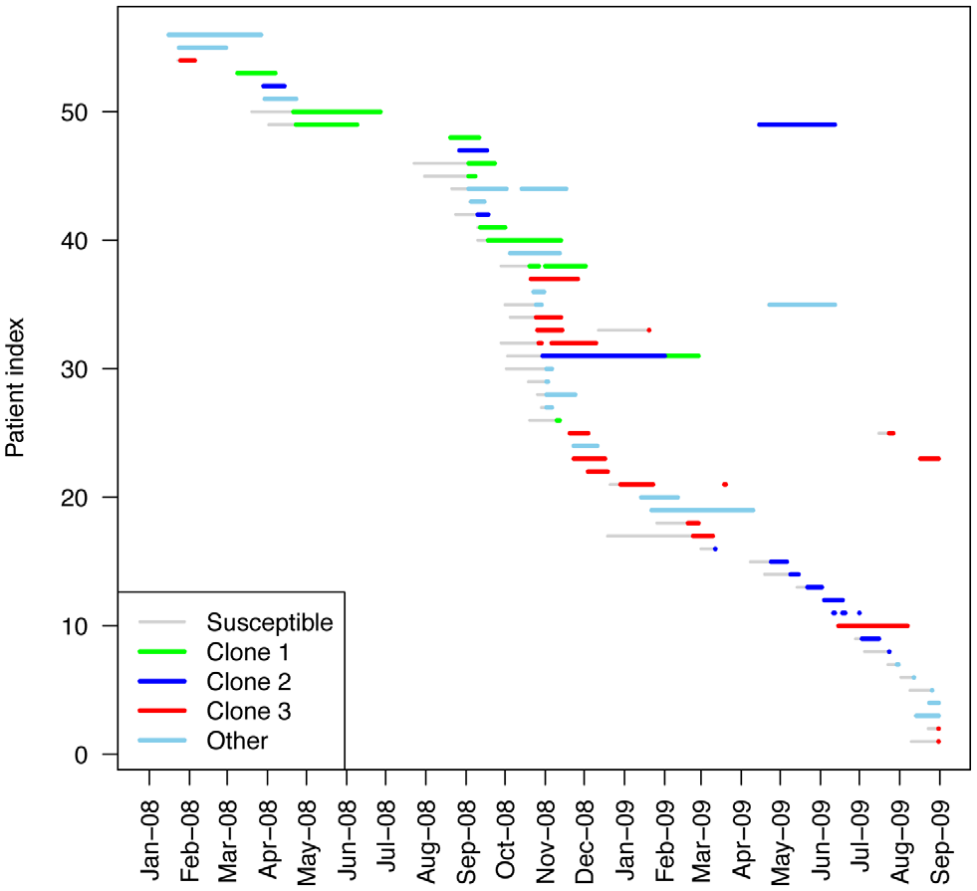
Patient episodes during the study period. Only those patients who had at least 1 MDRAB-positive swab are represented. Each patient’s stay is represented as a line, from admission to the first positive swab (grey line), and from first positive swab until discharge, with the color depending on the clone carried.

Results of cluster analysis of rep-PCR electrophoresis profiles are shown in Fig. 2. This analysis indicated 3 MDRAB clones spreading in the ward. Clone 1 included 14 isolates, with at least 85.6% homology. Clone 2 comprised 13 isolates, with at least 80.0% homology. Clone 3 consisted of 14 isolates, with a minimum homology of 90.7%. Finally, 15 nonclonal isolates could not be assigned to any cluster. Resistance profiles indicated that most isolates were resistant or intermediate to ceftazidim, ciprofloxacin and aminoglycosides, and susceptible to imipenem and colistin. No differences in the resistance profiles were detected between the clones. Fig. 3 shows a temporal picture of patients’ colonization episodes with clonal information and Fig. 1 the monthly prevalence for each clone. While clone 1 dominated early, clone 3 predominantly spread during the second part of the outbreak (October to December 2008). Clone 2 was observed almost throughout the period. All three clones were concomitantly observed during the outbreak.

**Figure 4:**
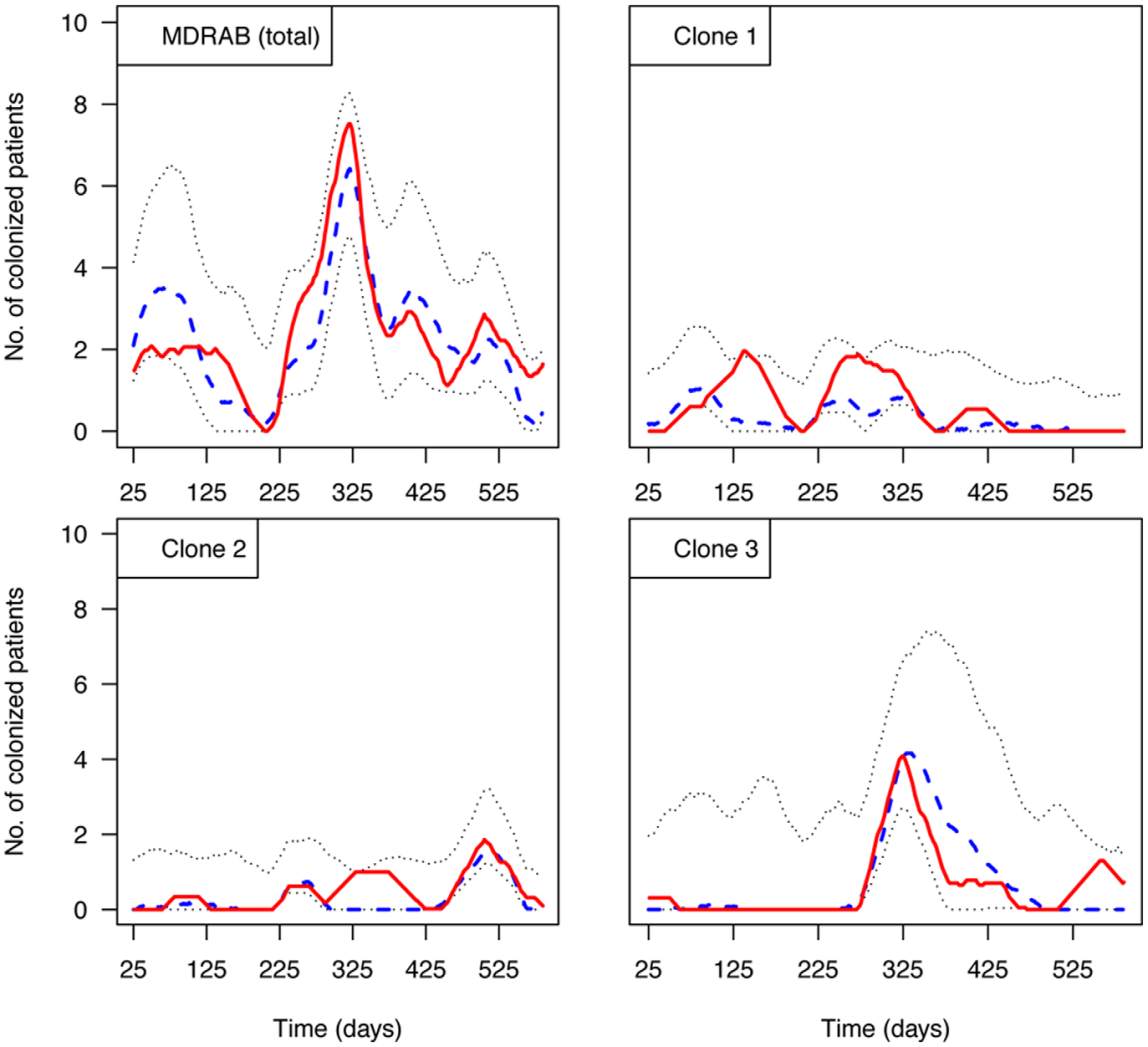
Model assessment. 50-day moving averages of the number of carriers of MDRAB or each MDRAB clone are represented for observations (red solid line), mean predicted values (blue dashed line) and 95% predicted intervals (black dotted lines).

Confronting the clonal transmission model with the data, we found that clone 3 had the highest transmissibility and the highest single-admission reproduction number. Although clones 1 and 2 had comparable transmissibilities, clone 1 had an intermediate single-admission reproduction number, while that of clone 2 was the lowest (Table 2). However, differences of transmissibility between clones were not significant, reflecting uncertainties in parameter estimates. For example, comparing clone 3 to all other strains, the estimated difference in transmission rates was 0.030 [-0.006–0.074] per day (p-value: 0.06). The respective estimated proportions of clone 1, 2 or 3 carriers at admission were 0.02 [0.01–0.05], 0.03 [0.01–0.06] and 0.03 [0.01–0.06].

To assess the model’s ability to predict the epidemic’s time evolution, we used the fitted model to generate 1,000 simulated epidemic curves for MDRAB and each MDRAB clone. These predictions were then compared to the observed data (see Material and Methods). For the nonclonal model, model assessment suggested a good fit to data before and after the outbreak. During the outbreak, the number of MDRAB-colonized patients tended to be underestimated, even though observations remained within the range of model predictions. Assessments for the clonal model also gave good fit to the data, but predicted intervals for clone 3 were wide, due to the large estimation interval for the transmissibility of this clone (Fig. 4).

## Discussion

Herein, we described a model-based approach to investigate the spread of multiple bacterial clones in a hospital ward. When clonal information is available, in addition to standard surveillance data for patients, e.g. from cluster analysis of pulse-field gel electrophoresis (PFGE) or rep-PCR profiles of isolates, the model provides a means to estimate clone transmissibility, which is measured by the transmission rate in the model. We applied this method to determine the MDRAB and genotype-specific MDRAB-epidemic abilities in a hospital setting. Our results also suggested enhanced transmissibility of one clone, although the difference was not significant.

Increasing trends of bacterial resistance have reemphasized the need for efficient control measures within hospitals and approaches to limit outbreaks, associated with a major public health impact. In this context, insights provided by mathematical models have gained considerable attention in the past few years [22], [23]. Recently, some methods were proposed to estimate the transmissibility of important nosocomial pathogens, a key parameter for hospital epidemiology and risk management. These methods were based on Markov [18], [24], or Bayesian hierarchical models [19], [25]. However, none of those studies included genotyping information, even when available [19].

Compared to those methods, ours has the advantage of incorporating clonal information directly into a mechanistic transmission model. The Markov chain formulation allows capture of most of the chance effects, an essential component when dealing with small populations. A possible limitation of this approach is that it requires extensive calculations as the model becomes more complex [26]. However, we circumvented this difficulty by adopting a one-versus-all strategy. As a consequence, regardless of the number of identified clones, the number of model states remains constant, thus allowing efficient and rapid calculations.

The method we used has a series of limitations that need to be addressed. First, MDRAB can survive for long periods in the environment, which then serves as a secondary source of transmission [27]. However, environmental samples taken during the outbreak returned negative, ruling out the possibility of an environmental contamination in this specific outbreak investigation. Therefore, we did not include a parameter representing an environmental reservoir in the final model. Other sources of acquisitions, not related to cross-transmission (e.g. endogenous colonization from a patient’s own flora), have been described and estimated in previous models [18], [28]. In a first version of the model, we included a parameter describing such sources but found no evidence that it was significant in our setting.

Regarding the molecular fingerprinting of isolates, rep-PCR was used instead of PFGE, which is viewed as the gold-standard method for molecular epidemiology. However, recent reports indicated that rep-PCR was a reliable and fast molecular-fingerprinting method for hospitals and had satisfactory resolution, compared to amplified fragment-length polymorphism and PFGE [16], [29], [30], [31]. With respect to the mathematical model, all patients were assumed to have the same risk of acquiring or transmitting MDRAB. Superspreader patients or healthcare workers have been described and modeled [32], but, considering the profile of patients in our setting, we think that the homogeneous population hypothesis was fair. More generally, as other modeling works in hospitals, several factors were omitted that could contribute significantly to the spread of MDRAB, such as staff workload, nursing organization, spatial proximity of patients, etc. More detailed models would be required to integrate those factors, but at the cost of being more difficult to estimate when little data is available. Once positive, patients were assumed to remain so until their discharge and antibiotic exposure was not taken into account in the model. Indeed, our data showed that MDRAB-colonized patients invariably remained so until their discharge. Isolated strains were all multiresistant, which might, in part, explain that observation. Finally, the MDRAB-screening test was assumed to be perfectly sensitive and specific.

The method described can be used to identify the most transmissible clones in a ward, using standard surveillance data. Although we restricted our analysis to a single unit, the method can easily be extended to work on a larger scale, e.g. several units or a hospital. Doing so would provide more global information on the transmissibility of MDRAB lineages, and also lead to follow-up studies dissecting the functional basis for variation in transmissibility.

On the other hand, there are currently several limits to apply this approach to inform infection control procedures. As a matter of fact, this study used data in a post hoc analysis, and standard control measures were used to manage the outbreak. The main drawback is the amount of time required to type isolates. However, rapid developments in typing technologies will dramatically reduce this delay in the near future, so that an online monitoring of an outbreak, combining results of molecular typing and mathematical modeling, could become possible. A future application of this work, consequently, would be to refine transmission-based precautions, based on early estimates of transmissibility for the spreading pathogens. Our method is also applicable to other nosocomial pathogens, as long as relevant epidemiological information is added to the model. Indeed, it is reasonable to expect comparable findings for other bacterial species, as was recently demonstrated for ST398 and non ST398 MRSA clones in Dutch hospitals [33].

Further studies are needed to explore the possibility that some clones are more epidemic than others. With respect to the *Acinetobacter baumannii* species, its epidemiology is characterized by the diffusion of a few widespread international clones often responsible for multidrug-resistant hospital outbreaks in many countries [15], [34]. Recent attempts to identify features of those clones, such as resistance to disinfection [35] or adherence to human cells [36], have so far failed to distinguish them from other genotypes. Thus, antimicrobial resistance seems to be the main evolutionary advantage accounting for their success. Moreover, enhanced ability of these clones to colonize human hosts would have exposed them to increased levels of antimicrobials. Our results possibly suggest the existence of different clonal epidemicities, which might be a good way to distinguish between successful and nonsuccessful clones.

With the advent of high-throughput sequencing technologies, genetic information on bacterial isolates will increasingly become available in hospital laboratories. In particular, whole-genome sequencing data have already been used to reveal worldwide routes of transmission [37], and will certainly have a great impact on healthcare epidemiology.

We conclude that the method and results presented herein could be relevant for dealing with these data and identifying possible threatening clones.

## Supporting Information

Text S1 Formulation of the transmission models and details of the estimation procedure.
(DOC)
