# Supplementary material for: Identifying More Epidemic Clones during a Hospital Outbreak of Multidrug-Resistant Acinetobacter baumannii
Source: PLoS One. 2012 Sep 27;7(9):e45758. doi: 10.1371/journal.pone.0045758 (PMC3459964; doi:10.1371/journal.pone.0045758)
Supplement: Text S1 — Formulation of the transmission models and details of the estimation procedure. (DOC) [file pone.0045758.s001.doc]

**Text s1**

**1. Formulation of the nonclonal transmission model.** At time *t*, we denote by *C(t)* the number MDRAB-colonized patients, *S(t)* the number of patients susceptible of acquiring MDRAB and *N = S(t) + C(t)* the total number of patients in the ward, taken to be constant. Patients enter the unit either MDRAB-negative, with probability *1 - σ*, or already MDRAB colonized, with probability *σ*. Susceptible patients were discharged at a rate *μ*, while colonized patients were discharged at a rate $\mu'$. Susceptible patients are at risk of acquiring MDRAB from other colonized patients (via contaminated health-care workers), at rate *βC(t)/N* per day, where *β* is the transmission rate. Patients positive for MDRAB carriage were assumed to remain so until their discharge and to be able to transmit their pathogen instantaneously to other patients. Given these assumptions, the deterministic equations of MDRAB-transmission dynamics in the ward can be written:

Because the study population is small (~15 patients), it is essential that a stochastic formulation of this model be used. A good way to do so is to derive a continuous-time Markov process from these equations, using master equations . Let *p_i_(t)* be the probability of having exactly *i* MDRAB-colonized patients in the ward, at time *t*. Then, the master equations, describing the temporal dynamics of the distribution of states, for *i* in [0,*N*], are:

where *Q* is the matrix of transition rates, and ***p****(t)* the vector representing the distribution of states at time t. The exact solution for this system is given by:

Given a set of exact observations (*x_1_*, *x_2_*, …, *x_n_*) made at time *t_1_*, *t_2_*, … , *t_n_*, it is possible to write a likelihood function, quantifying the probability of observing the data, given the model:

**2. Formulation of the clonal transmission model.** Here, the number of model states depends on the number of clones identified by molecular typing. Because the number of transitions in the model grows exponentially with the number of states, inference for a complete model soon becomes intractable . Instead, we adopted the following one-versus-all strategy. The number of clones identified by molecular fingerprinting is denoted *n*. For each *k*, *k* = 1, …, *n*, we denote by *C_k_(t)* the number of clone *k*-colonized patients at time *t* and *C_–k_(t)* the number of patients colonized with a strain not belonging to clone *k* at time *t*, so that *C_–k_(t) + C_k_(t) = C(t)*. Once colonized with a clone, it is still assumed that each patient remains so until discharge and, furthermore, that patients colonized with a clone can no longer acquire another MDRAB strain. Again, the set of deterministic equations, describing the transmission dynamics of each clone in the ward, can be written as:

where *σ_k_*, *μ_k_*, *β_k_* (or *σ_–k_*, *μ_–k_*, *β_–k_*) are, respectively, the importation probability, discharge rate and transmission rate for patient colonized with clone *k* (or patients colonized with a strain not belonging to clone k), and *μ* the discharge rate for susceptible patients.

As before, a continuous-time Markov process was derived, taking into account the stochasticity of the process. Model-state distributions can be computed as and a likelihood function can be written, if exact observations are made at regular times.

**3. Markov Chain Monte Carlo (MCMC) algorithm for parameter estimations**

We used a previously described method based on data augmentation techniques to estimate model parameters .

***3.1. Updating the parameters.*** In our implementation of the MCMC algorithm, we updated all estimated parameters in batch, and then updated the augmented data. $\theta$denotes the vector of parameters to be estimated. For the nonclonal transmission model,$\theta=(\beta,\sigma)$, whereas for the clonal transmission model,$\theta=(\beta_{k},\beta_{-k},\sigma_{k},\sigma_{-k})$. A proposal $\theta_{i}^{*}$for the new value of the *i*^th^ element of $\theta$ is chosen by drawing from the proposal distribution, $J_{i,c}(\theta_{i}^{*}|\theta_{i,c})\sim LN(\ln(\theta_{i,c}),\sigma^{2}_{\theta_{i}})$, where $\theta_{i,c}$ is the current value of $\theta_{i}$ and $\sigma_{\theta_{i}}^{2}$the proposal variance for $\theta_{i}$. The proposed values for each $\theta$component are then accepted with a probability $r_{\theta}$, chosen to ensure the chain has the correct stationary distribution:

$$r_{\theta}=\min(1,\frac{p(D|\theta^{*})p(\theta^{*})J_{c}(\theta_{c-1}|\theta^{*})}{p(D|\theta_{c-1})p(\theta_{c-1})J_{c}(\theta^{*}|\theta_{c-1})})$$

where $p(D|\theta)$is the likelihood function and $p(\theta)$is the prior density of $\theta$.

***3.2. Updating the augmented data.*** $D_{c}$denotes the current augmented data. A patient whose exact colonization date is uncertain is randomly selected in the dataset. A proposed colonization time is then uniformly selected among the days the patient could have feasibly acquired the strain. Let $D^{*}$represent the proposed augmented data, in which this patient’s colonization date was modified. A decision to accept this move is made with probability $r_{D}$:

$$r_{D}=\min(1,\frac{p(D^{*}|\theta)}{p(D_{c}|\theta)})$$

References

1. Keeling MJ, Ross JV (2008) On methods for studying stochastic disease dynamics. J R Soc Interface 5: 171–181.

2. Cooper BS, Medley GF, Bradley SJ, Scott GM (2008) An augmented data method for the analysis of nosocomial infection data. Am J Epidemiol 168: 548–557.
